# Ectopic expression of *LONELY GUY7* in epidermis of internodal segments for *de novo* shoot regeneration without phytohormone treatment in ipecac

**DOI:** 10.1111/ppl.70023

**Published:** 2024-12-26

**Authors:** Karin Okazaki, Wataru Katano, Kyomi Shibata, Masashi Asahina, Kazuko Koshiba‐Takeuchi, Koichiro Shimomura, Mikihisa Umehara

**Affiliations:** ^1^ Graduate School of Life Sciences, Toyo University Asaka‐shi Saitama Japan; ^2^ Department of Biosciences Teikyo University Utsunomiya Tochigi Japan; ^3^ Advanced Instrumental Analysis Center. Teikyo University.1‐1 Toyosatodai Utsunomiya Tochigi Japan; ^4^ Present address: Present Address: Division of Molecular Cardiovascular Biology Cincinnati Children's Hospital Medical Center Cincinnati OH USA

## Abstract

In many plant species, the application of exogenous phytohormones is crucial for initiating *de novo* shoot regeneration. However, ipecac [*Carapichea ipecacuanha* (Brot) L. Andersson] has a unique ability to develop adventitious shoots on the epidermis of internodal segments without phytohormone treatment. This characteristic allows us to evaluate the effects of endogenous phytohormones in this species. Here, we showed that the presence of the pith, including vascular bundles in the internodal segment, is required to activate both endogenous cytokinin (CK) biosynthesis and adventitious shoot formation. Adventitious shoots were mainly formed in the apical region of internodal segments, where the CK biosynthesis genes *ISOPENTENYL TRANSFERASE 3* (*CiIPT3*) and *LONELY GUY 7* (*CiLOG7*) were spontaneously upregulated in the early culture stage on phytohormone‐free medium. In addition, *CiIPT3* and *CiLOG7* were respectively expressed in the pith and the epidermis of the internodal segments. The expression of *CiLOG7* was localized as several spots on the epidermis. These findings suggest that CK precursors are generated in the pith, transferred to the epidermis, and then converted into active CKs, facilitating adventitious shoot formation on the epidermis. Conversely, auxin levels rapidly decreased during culture and remained low in the region of shoot formation. Auxin is transferred to the basal region of internodal segments, and strongly suppressed the *CiLOG7* expression and decreased the CK levels*.* Thus, we conclude that the ectopic expression of *CiLOG7* in the epidermis of internodal segments contributes to *de novo* shoot regeneration in ipecac.

## 1 INTRODUCTION

Plant cells have high plasticity and are able to regenerate the whole plant body through *de novo* organogenesis (Sang et al. 2018). Auxin and cytokinins (CKs) are the principal regulators of this organogenesis. In tobacco, adventitious roots are artificially induced from explants on auxin‐rich medium, while adventitious shoots are induced in CK‐rich medium (Skoog and Miller 1957). Plant tissue culture systems have been established to regenerate the whole plant body from explants isolated from mother plants on culture media containing auxins and CKs at optimum combinations and concentrations in many plant species (Ikeuchi et al. 2019). In the model plant *Arabidopsis thaliana*, plant regeneration occurs in two steps: first, pluripotent callus is formed on auxin‐rich callus‐inducing medium, and shoots then differentiate from the callus on CK‐rich shoot‐inducing medium (Valvekens et al. 1988, Duclercq et al. 2011a). Owing to CK overproduction, the *Arabidopsis hoc* mutants have a high shoot regeneration capacity on culture medium with no exogenous CKs (Catterou et al. 2002, Duclercq et al. 2011b). The shoot regeneration frequencies are lower in *Arabidopsis atipt3 atipt5 atipt7* triple mutants than in the wild type (Cheng et al. 2013). Overexpression of a CK biosynthetic gene increases shoot regeneration in tobacco and lettuce (Kunkel et al. 1999). In these cases, an increase in endogenous CKs plays an important positive role in plant regeneration.

To increase endogenous CK levels, CK biosynthesis is needed to be activated. The major CK biosynthesis pathway begins with the formation of *N* ^6^‐(Δ^2^‐isopentenyl)adenine (2iP) ribotides through the transfer of a prenyl moiety from a dimethyl allyl pyrophosphate to the *N* ^6^ position of ATP or ADP by adenosine phosphate–isopentenyl transferases encoded by *ISOPENTENYL TRANSFERASE* (*IPT*) (Kakimoto 2001, Takei et al. 2001). CYP735As (CK hydroxylase) hydroxylate the prenyl moiety of the 2iP ribotides, resulting in the formation of *trans*‐zeatin (tZ) ribotides (Takei et al. 2004). A nucleotide 5′‐monophosphate phosphoribohydrolase encoded by *LONELY GUY* (*LOG*) converts both 2iP and tZ ribotides to the bioactive CKs 2iP and tZ, thus playing a pivotal role in regulating CK activity (Kurakawa et al. 2007, Kuroha et al. 2009). The bioactive CKs are perceived by the CK receptors ARABIDOPSIS HISTIDINE KINASE 2 (AHK2), AHK3, and AHK4 (Inoue et al. 2001, Suzuki et al. 2001, Yamada et al. 2001). The CK signal is transmitted via histidine phosphotransfer proteins to the B‐type transcription factors ARABIDOPSIS RESPONSE REGULATORs (ARRs) (Hwang and Sheen 2001). CK signaling via ARR1, ARR2, ARR10, and ARR12 directly enhances the expression of *WUSCHEL*, which encodes a homeodomain transcription factor and functions as a key regulator in the formation of shoot apical meristem (Meng et al. 2017, Zhang et al. 2017).

Interestingly, wild‐type plants of several species can form adventitious shoots on explants without phytohormone treatment. Ipecac [*Carapichea ipecacuanha* (Brot.) L. Andersson] can form adventitious shoots on internodal segments in culture medium without exogenous auxins and CKs (Yoshimatsu and Shimomura 1991). This characteristic allows us to analyze changes in endogenous auxin and CK levels during adventitious shoot formation and to evaluate the effects of endogenous phytohormones in this species. Adventitious shoots are formed on the apical region of internodal segments but not on the basal region (Koike et al. 2017). Primordia of these adventitious shoots are derived from epidermal cells and are absent at the cut end of internodal segments. Exogenous auxin strongly inhibits adventitious shoot formation in ipecac, and endogenous levels of indole‐3‐acetic acid (IAA) are low in the apical region of internodal segments and high in the basal region. IAA is transported from the apical to basal region during culture, so its concentration remains low in the apical region (Koike et al. 2020). These data indicate a negative relation between the position of the adventitious shoots formed and IAA distribution in internodal segments. Additionally, exogenous strigolactone inhibits adventitious shoot formation in ipecac, while inhibitors of strigolactone biosynthesis and signaling stimulate it because they enhance the expression of *ENHANCER OF SHOOT REGENERATION 2* (Okazaki et al. 2021, Okazaki et al. 2023).

In ipecac, endogenous CKs transiently increase in the internodal segments during culture (Koike et al. 2017), and CK biosynthesis genes (*CiIPT3* and *CiLOG7*) were upregulated in the apical region of internodal segments before adventitious shoot formation (Okazaki et al. 2022). We hypothesized that CK biosynthesis plays an important role in adventitious shoot formation in ipecac. In this study, we investigated how the expression of CK biosynthesis genes is regulated during adventitious shoot formation in ipecac. First, we vertically cut the internodal segments into two sections (internodes with or without the pith including vascular bundles) and cultured them. Second, we analyzed the tissue‐specific expression of *CiIPT3* and *CiLOG7* by laser microdissection, examined the dynamics of endogenous CK levels by mass spectrometry, and determined the localization of *CiLOG7* mRNA by *in situ* hybridization. Further, we investigated the effects of exogenously applied auxin on CK biosynthesis in the internodal segments.

## 2 MATERIALS AND METHODS

### 2.1 Plant materials and culture conditions

Ipecac [*Carapichea ipecacuanha* (Brot.) L. Andersson] plants are continuously maintained under sterile conditions at Toyo University. Sterile plants were dissected into shoot tips, nodes, and internodes, which were then placed on 25 mL phytohormone‐free B5 culture medium (Gamborg et al. 1968) solidified with 0.2% Gelrite in a Petri dish (internal diameter, 90 mm; height, 20 mm). The plants were cultured at 24°C under a 14‐h light / 10‐h dark photoperiod (10–20 μmol photons m^−2^ s^−1^) for approximately 2 months. The method for ipecac tissue culture was established by Yoshimatsu and Shimomura (1991). Internodal segments were cut to 5 mm in length in most experiments and placed horizontally on B5 culture medium to induce adventitious shoots. Adventitious shoots longer than 0.3 mm were counted under a digital microscope (DHS1000; Leica Microsystems). Each experiment was repeated three or four times.

### 2.2 Chemicals

*N* ^*6*^‐(Δ^2^‐isopentenyl)adenine (2iP) and *trans*‐zeatin (tZ) were purchased from Sigma‐Aldrich, isopentenyl adenine riboside (iPR) was from Kanto, and *trans*‐zeatin riboside (tZR), indole‐3‐acetic acid (IAA) and 1‐naphthylacetic acid (NAA) were from Fujifilm Wako Pure Chemical Industry Corporation. 4‐chloro‐α‐(phenyl ethyl‐2‐one)‐IAA (4‐Cl‐PEO‐IAA) was provided by Prof. Ken‐ichiro Hayashi (Okayama University of Science, Japan). 2iP, tZ, and NAA were dissolved in water, and 1 mM stocks were stored at −30°C; they were sterilized by filtration and added to B5 culture medium after autoclaving. 4‐Cl‐PEO‐IAA was dissolved in acetone, and 10 mM stock was stored at −30°C. Deuterium‐labeled phytohormones (D_6_‐2iP, D_6_‐iPR, D_5_‐tZ, D_5_‐tZR, and D_5_‐IAA) were purchased from OlChemIm and used as internal standards in the liquid chromatography–tandem mass spectrometry (LC–MS/MS) analysis. The standards were dissolved in acetonitrile to prepare 100 pg. μL^−1^ stock solutions, which were used to generate standard curves.

### 2.3 Sudan Red staining

Sudan red staining was performed according to a previously published paper (Kadokura et al. 2018). Sudan Red 7B was purchased from Sigma‐Aldrich. Internodal segments after 5 weeks of culture were dehydrated through a series of isopropanol solutions (20%, 40%, and 60% v/v) and then incubated for 1 hour in 0.5% (w/v) Sudan red 7B in 60% (v/v) isopropanol. The segments were subsequently rehydrated in a series of isopropanol (60%, 40%, and 20% v/v). Afterward, the segments were washed three times with ultra‐pure water. Samples were observed under a digital microscope (DHS1000; Leica Microsystems). Seeds of *Arabidopsis thaliana* (Col‐0) were used as a positive control for the staining.

### 2.4 Gene expression analysis

Internodal segments were collected in a 2.0‐mL tube with a zirconia bead (diameter, 5 mm), frozen in liquid nitrogen and crushed in a TissueLyser II (Qiagen). Total RNA was extracted with an RNeasy Plant Mini Kit (Qiagen), and cDNA was synthesized from 400 ng of total RNA using ReverTra Ace qPCR RT Master Mix (Toyobo). qRT‐PCR was performed using a Thunderbird NEXT SYBR qPCR Mix kit (Toyobo) in a LightCycler 96 System (Roche Diagnostics) as follows: 95°C for 20 s; 40 cycles of 95°C for 3 s, and 60°C for 30 s; and a final 5‐min extension at 72°C in the cycling stage, followed by 95°C for 15 s, 60°C for 1 min, and 95°C for 15 s in the melt‐curve stage. *Carapichea ipecacuanha ELONGATION FACTOR1* (*CiEF1*) was used as an internal standard. The primer sets are listed in Table S1.

### 2.5 Analysis of endogenous CKs and IAA

Internodal segment samples were prepared for gene expression analysis and were suspended in 1 mL acetonitrile containing 1% (v/v) acetic acid, 200 pg. of each D_6_‐2iP, D_6_‐iPR, D_5_‐tZR, and D_5_‐IAA; and 400 pg. D_5_‐tZ. The samples were incubated for 1 h at 4°C in the dark and centrifuged at 3,500 × *g* for 5 min at room temperature. The precipitate was washed with 1 mL of 80% (v/v) acetonitrile containing 1% (v/v) acetic acid and centrifuged again. Both supernatants were combined and 600 μL 1% (v/v) acetic acid in water was added. Acetonitrile in the sample was evaporated by nitrogen gas flow, and the aqueous sample was loaded onto an Oasis MCX cartridge column (Waters) equilibrated in 1% (v/v) acetic acid. The column was washed with 1% (v/v) acetic acid, and IAA was eluted with 2 mL of 30% (v/v) acetonitrile containing 1% (v/v) acetic acid. The column was washed consecutively with 2 mL of 80% (v/v) acetonitrile containing 1% (v/v) acetic acid, 1 mL of water, and 1 mL of 5% (v/v) NH_3_ in water. CKs were eluted with 2 mL of 60% (v/v) acetonitrile containing 5% (v/v) NH_3_ in water. All IAA and CK fractions were evaporated and subjected to LC–MS/MS analysis using a triple‐quadrupole MS system (3200 QTRAP; Sciex) and an HPLC system (Prominence). Parameters for LC–MS/MS analysis were described in our previous article (Koike et al. 2018). An Acquity HSS T3 UPLC column was used for CK analysis and an Acquity BEH C18 UPLC column for IAA analysis (both columns: diameter, 2.1 mm; height, 50 mm; Waters).

### 2.6 Laser microdissection

Internodal segments were collected in a 1.5‐mL tube and frozen in liquid nitrogen. Cryo‐sections were prepared according to a previously published paper (Notaguchi et al. 2020). Frozen segments were sliced at 15‐μm thickness at −25°C in a cryo‐chamber of a CM1860 cryostat (Leica Biosystems). Three sections were excised (pith, cortex, and epidermis) using laser microdissection (LMD7000; Leica Microsystems) and collected into RNA extraction buffer composed of buffer RLT (Qiagen) supplemented with 0.01% β‐mercaptoethanol. Total RNA was extracted with an RNeasy Plant Micro Kit (Qiagen), and cDNA was synthesized from 1–33 ng of total RNA using ReverTra Ace qPCR RT Master Mix (Toyobo). qRT‐PCR was performed as described above, and expression was normalized to that in the pith after 2 days of culture.

### 2.7 *In situ* hybridization

Preparation of paraffin sections and *in situ* hybridization were performed according to previous papers (Nakamura et al. 2016, Koshiba‐Takeuchi 2018). *CiLOG7* probes (654 bp) were designed from partial sequences detected in our RNA‐seq analysis (Okazaki et al. 2022). Partial *CiLOG7* cDNA for the probe template was amplified by PCR, and antisense and sense probes were synthesized using T3 and T7 RNA polymerases, respectively. Internodal segments were fixed in formaldehyde/acetic acid (FAA) solution (formalin: acetic acid: 50% ethanol = 1:1:18 v/v/v) at 4°C for 1 day. Paraffin sections were sliced at 10‐μm thickness on an RM2245 microtome (Leica Biosystems). DIG‐labeled probes were hybridized overnight at 45°C. DIG‐labeled mRNA was detected using BM Purple and observed under a BZ‐9000 microscope (Keyence).

### 2.8 Preparation of cryo‐sections

Cryo‐sections were prepared according to our previous work (Koike et al. 2017). Internodal segments were fixed in FAA solution at 4°C for 1 day, washed with Milli‐Q water to remove the fixative, and placed in 20% sucrose solution for 1 day. Cryo‐sections were cut at 5‐μm thickness at −20°C in a cryo‐chamber of a CM3050 S cryostat (Leica Biosystems) (Kawamoto 2003). The sections were stained with 0.05% toluidine blue *O* and observed under the BZ‐9000 microscope.

### 2.9 Statistical analysis

Statistical analysis was performed in IBM SPSS Statistics 28.0 software (IBM SPSS Inc.). Following assessment of the equality of variances by ANOVA, experimental groups were compared by Tukey's honestly significant difference (Tukey's HSD) test. *P‐*values less than 0.05 were considered statistically significant. Pairwise comparisons were performed by *t*‐test after evaluation of variance by *F*‐test. All experiments were carried out in a completely randomized design.

## 3 RESULTS

### 3.1 Pith of internodal segments is required for adventitious shoot formation on phytohormone‐free culture media

**FIGURE 1:**
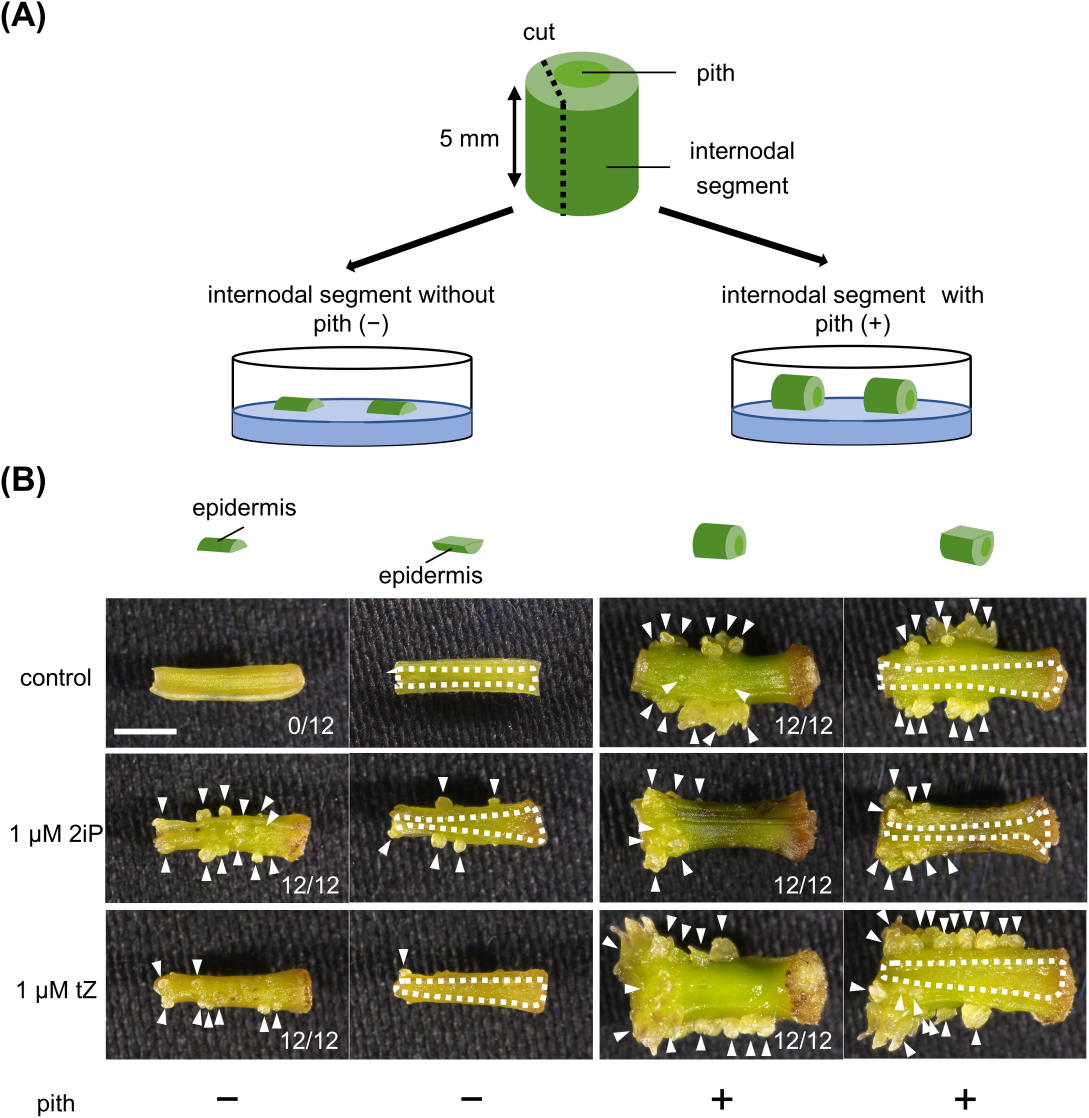
Effects of the pith on adventitious shoot formation in ipecac. (A) Division of internodal segments into sections with or without the pith including vascular bundles. (B) Representative images of tissues after 4 weeks of culture under the indicated conditions. White arrowheads, adventitious shoots; dotted lines, areas where epidermis was excised off. Number of internodal segments with adventitious shoots formed is shown in each image. The experiment was conducted twice, with six segments per experiment. 2iP, *N* ^6^‐isopentenyladenine. tZ, *trans*‐zeatin. Bar, 2 mm.

*De novo* shoots formed on internodal segments of ipecac were not stained by Sudan Red staining, whereas Arabidopsis zygotic embryos were stained (Figure S1). The shoots were therefore confirmed to be adventitious shoots. We cut ipecac internodal segments vertically or horizontally and varied the shape and tissue composition of the explants. To evaluate the effects of pith during adventitious shoot formation, we divided each internodal segment into two sections—one with and the other one without the pith including vascular bundles (Figure 1A)— and cultured the sections on a phytohormone‐free medium for 4 weeks. Adventitious shoots were formed on the epidermis of all the sections with the pith, except in areas where the epidermis was excised (Figure 1B). In contrast, no adventitious shoots formed on any sections without pith when cultured without phytohormones (Figure 1B). In the presence of 1 μM 2iP or tZ, several adventitious shoots were induced on the epidermis of the sections without pith, but not on the areas where epidermis was excised. After the experiment, the presence or absence of the pith was confirmed by examining cryo‐sections of the internodal segments (Figure S2).

To explore the impact of varying internode segment length on adventitious shoot formation, we cut the segments to lengths of 1, 2, 3, 4, or 5 mm (Figure S3A) and cultured them on a phytohormone‐free culture medium for 5 weeks. The number of adventitious shoots increased similarly in the 2 mm or longer internodal segments (Figure S3B), and the shoots were formed mainly on the apical regions (Figure S3C), indicating that the length of internodal segments did not significantly affect the number of adventitious shoots per explant.

### 3.2 Pith of internodal segments is required for endogenous CK production

We have revealed that the expression of CK biosynthesis genes (*CiIPT3* and *CiLOG7*) in internodal segments is higher after 7 days of culture than before culture (Okazaki et al. 2022). When internodal segments were divided into regions designated I to IV (from apical to basal; Figure S4), the expression of *CiIPT3* and *CiLOG7* was low before culture and was similar in all four regions. After 7 days of culture, *CiIPT3* and *CiLOG7* showed the highest expression in regions II and I, respectively (Figure S4). The expression of both genes was not upregulated in the basal region.

**FIGURE 2:**
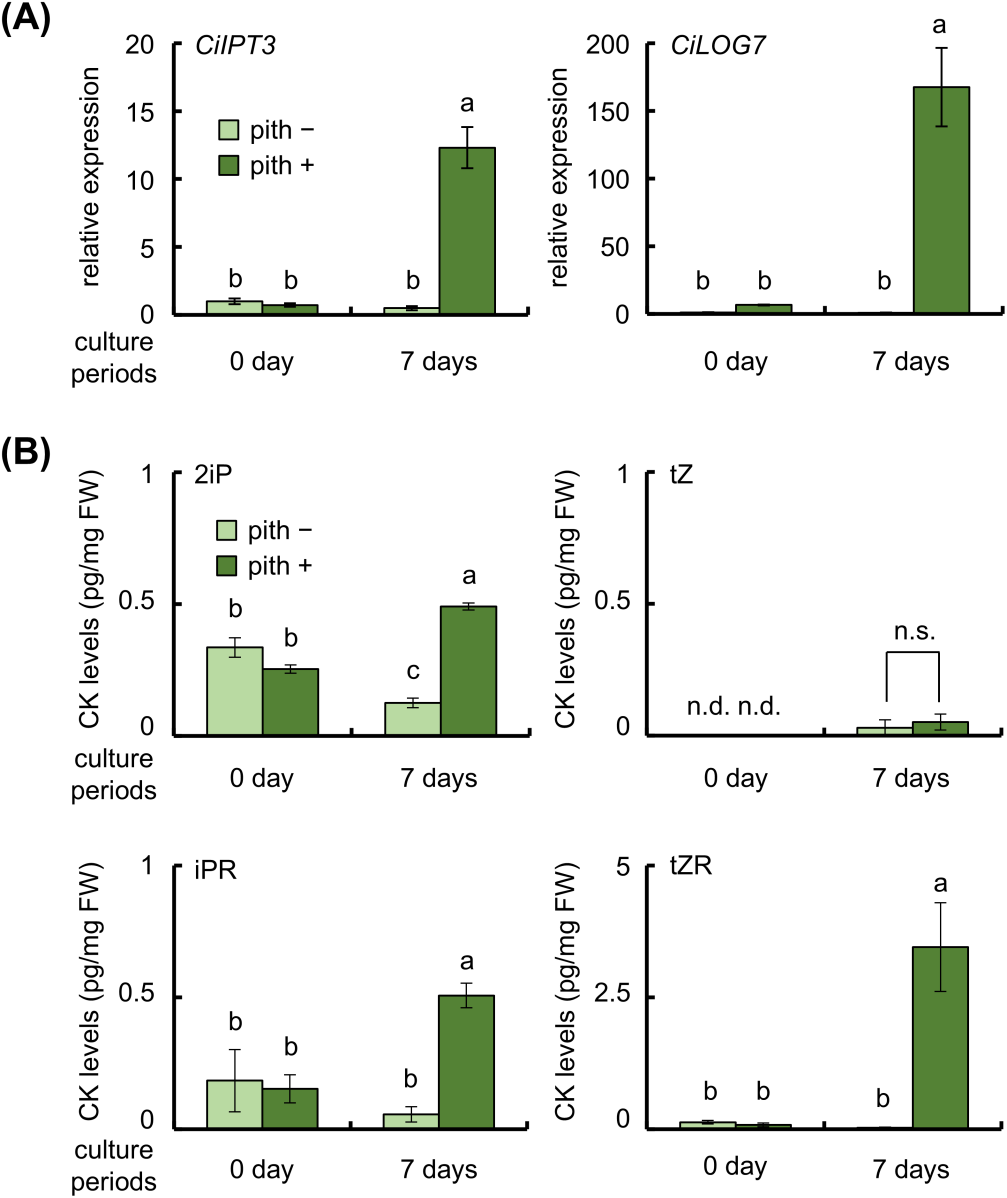
CK biosynthesis in internodal segments with or without the pith. Tissues were collected before culture (0 day) or after 7 days of culture on a phytohormone‐free culture medium. Internodal segments (4 with and 14 without the pith) were used in each experiment. (A) Relative expression of CK biosynthesis genes. *EF‐1* was used as an internal standard. The data were normalized to the expression in the internodal segment without a vascular bundle before culture. (B) Endogenous CK levels in internodal segments. 2iP, *N* ^6^‐isopentenyladenine. tZ, *trans*‐zeatin. iPR, isopentenyladenine riboside. tZR, trans‐zeatin riboside. Data are mean ± SE (*n* = 4). Different letters indicate significant differences among segments (Tukey's HSD, *p* < 0.05; *CiIPT3*, *CiLOG7*, 2iP, iPR, tZR) or between internodal segments with versus without the pith (Student's *t‐*test; tZ, all *p* > 0.05); n.d., not detected, n.s., not significant.

Next, we investigated how the expression levels of these genes change in internodal segments with or without the pith after 1 week of culture (Figure 2A). *CiIPT3* and *CiLOG7* expression in sections with the pith after 7 days of culture was 12 and 170 times that before culture, respectively. In contrast, there was no significant difference between before culture and after 7 days of culture in the expression of *CiIPT3* and *CiLOG7* in sections without the pith (Figure 2A).

Endogenous CK and IAA levels are higher in internodal segments after 7 days of culture than before culture (Koike et al. 2017). To investigate whether the pith affects CK levels, we measured endogenous levels of 2iP, iPR, tZ, and tZR in internodal segment sections by using LC–MS/MS (Figure 2B). Before culture, 2iP, iPR, and tZR were almost at the same levels in sections with and without the pith, whereas tZ levels were below the detection limit in both section types. After 7 days of culture, 2iP, iPR, and tZR levels in sections with the pith were 2, 3.3, and 43.3 times those before culture, respectively. On the other hand, they tended to decrease in sections without the pith. tZ became detectable after 7 days of culture, with no significant differences between sections with and without the pith (Figure 2B). IAA levels were significantly higher in sections with the pith than in those without the pith, and decreased after 7 days of culture (Figure S5A).

### 3.3 Time course analysis of CK biosynthesis genes and endogenous CKs

**FIGURE 3:**
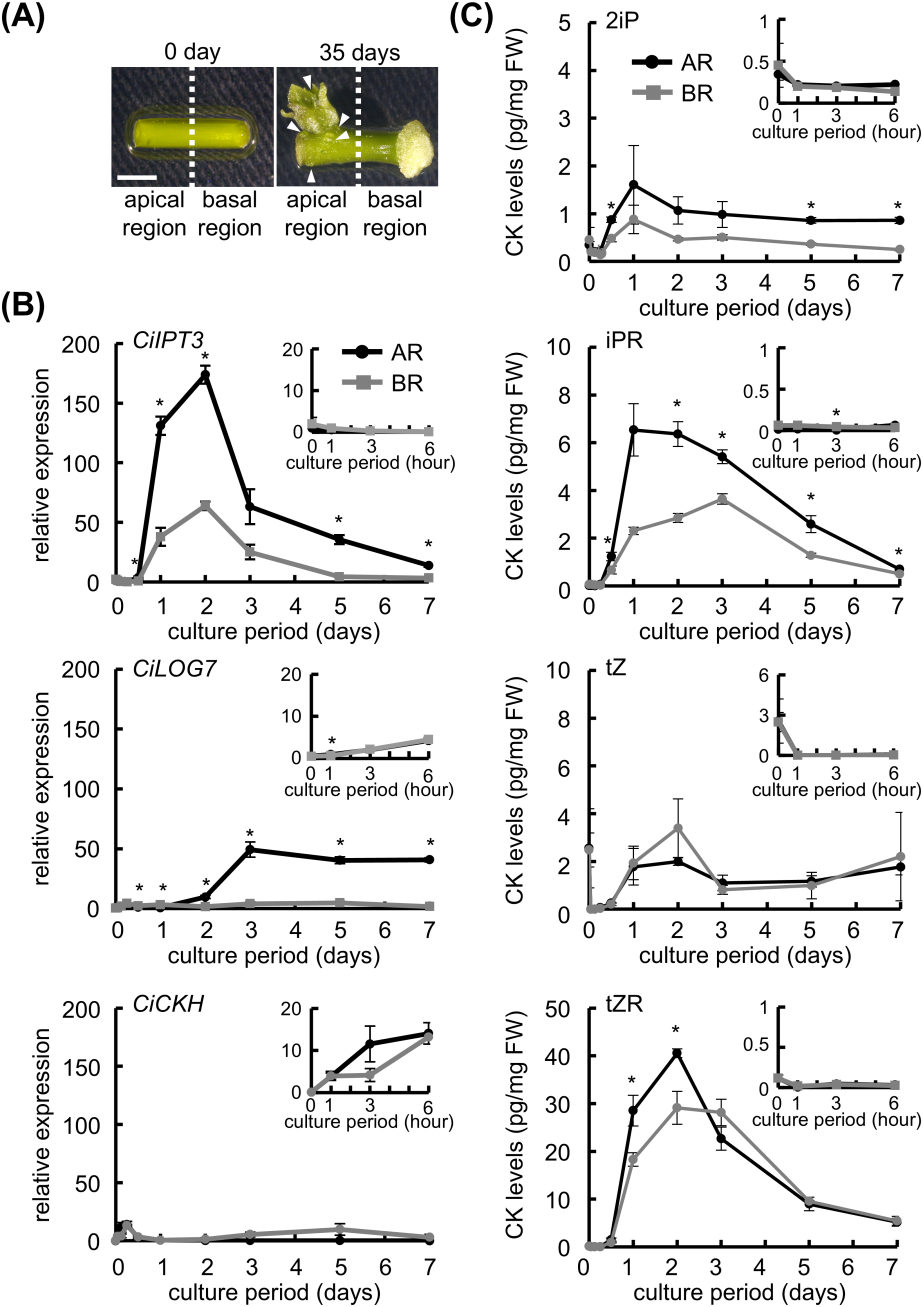
Time course analysis of CK biosynthesis in internodal segments during the initial 7 days of culture. (A) Representative images of tissues after 0 day and 35 days of culture on hormone‐free culture medium. White arrowheads, adventitious shoots. Bar, 2 mm. (B) Relative expression of CK biosynthesis genes. *EF‐1* was used as an internal standard. The data were normalized to the *CiIPT3* expression in the apical region before culture. Data are mean ± SE (*n* = 4). Internodal segments were collected before culture (0 day), after 1 h, 3 h, 6 h, 12 h, 1 day, 2 days, 3 days, 5 days, and 7 days of culture on phytohormone‐free culture medium. Then, the internodal segments were divided into the apical region and basal region sections. Five segments were used in each experiment. AR: apical region, BR: means basal region. (C) Endogenous CK levels in internodal segments. Insets show enlarged 0–6‐h plots. Four segments were used in each experiment. Data are mean ± SE (*n* = 3). Asterisk indicates significant difference compared with apical region (*t*‐test, *p* < 0.05).

In ipecac, adventitious shoots are formed on the apical region of internodal segments (Figure 3A). We examined temporal changes in the expression of *CiIPT3*, *CiLOG7*, and *CK hydroxylase* (*CiCKH*) in internodal segments during 7 days of culture on a phytohormone‐free medium (Figure 3B). Each segment was divided into the apical region and basal region. In both regions, *CiIPT3* expression increased significantly at 1 day of culture, peaked at 2 days, and then decreased gradually. It was higher in the apical region than in the basal region (2.7 times at 2 days). *CiLOG7* expression increased significantly at 3 days of culture in the apical region and remained high until 7 days, whereas expression in the basal region did not change significantly. *CiLOG7* expression at 3 days in the apical region was approximately 11.8 times that in the basal region. Expression of *CiCKH*, which is associated with tZ‐type‐CK biosynthesis, was very low compared with that of *CiIPT3* and *CiLOG7* in both apical and basal regions of internodal segments. CK metabolism may interact with excessive CK production to maintain CK homeostasis. Thus, we performed an expression analysis of *CYTOKININE OXIDASE 7* (*CiCKX7*). *CiCKX7* expression increased significantly, peaked at 2 days of culture, and then gradually decreased (Figure S6).

We performed a time course analysis of endogenous CK and IAA levels in the apical and basal regions during the initial 7 days of culture (Figure 3C). After the culture, internodal segments were divided into two segments (apical and basal regions). The 2iP level increased significantly in the apical region at 1 day of culture, decreased slightly at 2 days, and then remained at a similar level. That of iPR increased significantly at 1 day in the apical region and then decreased gradually. Those of tZ and tZR slightly decreased at 1 hour of culture, increased at 1 day of culture and peaked at 2 days. 2iP‐type CK levels tended to be higher in the apical region than in the basal region, whereas tZ‐type CK levels showed no clear difference between these regions. The IAA level in the apical region decreased significantly after 1 h of culture and remained low, whereas that in the basal region decreased at 3 hours and increased gradually after 12 hours (Figure S5B).

### 3.4 Localization of mRNA of CK biosynthesis genes in internodal segments

**FIGURE 4:**
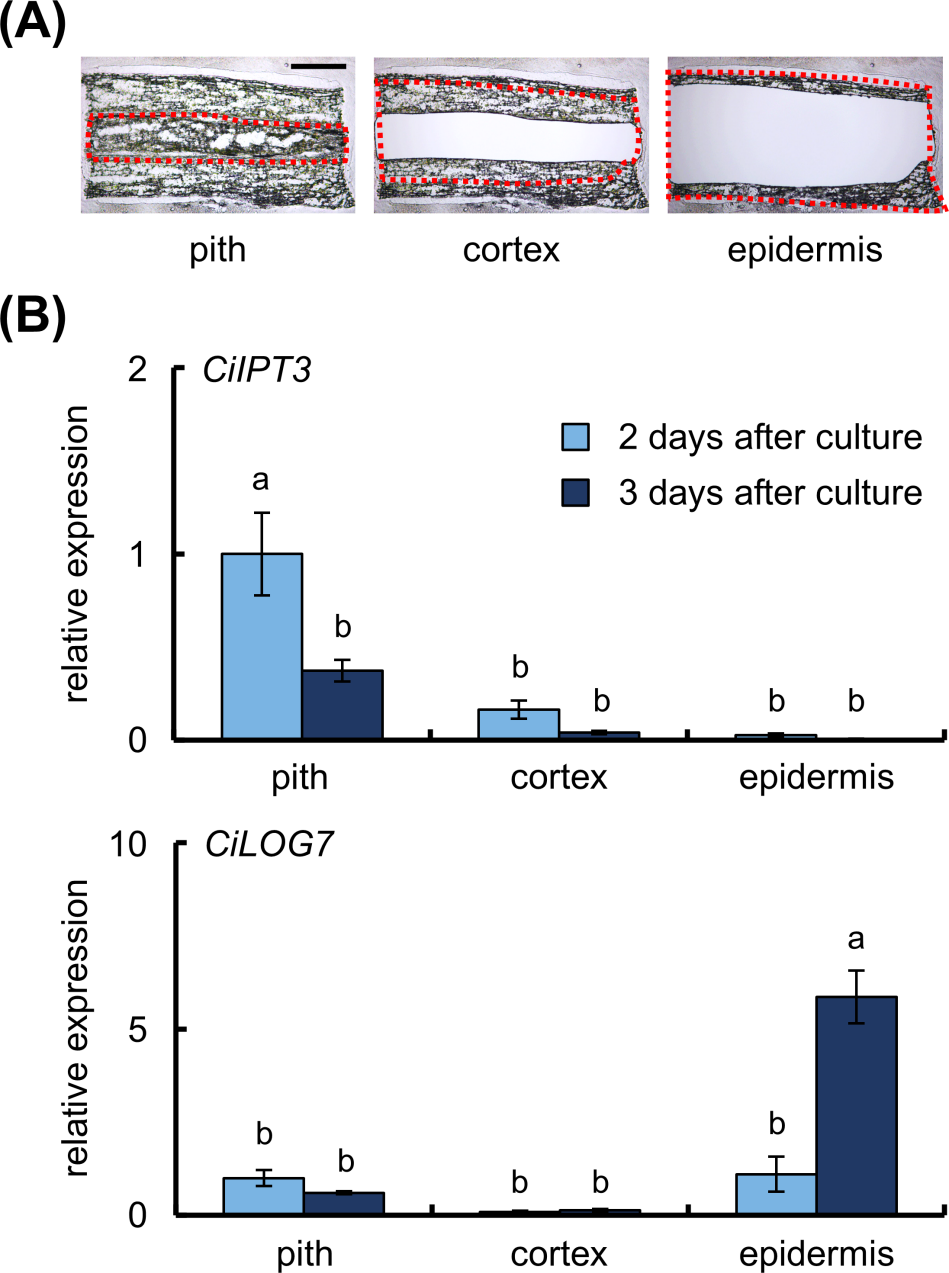
Relative expression of CK biosynthesis genes in the pith, cortex, and epidermis of internodal segments. (A) Cryo‐sections after laser microdissection. Red dotted lines indicate the area excised by laser microdissection. Bar, 500 μm. (B) Relative expression of *IPT3* and *LOG7* in each section. Five segments were used in each experiment. Data are mean ± SE (n = 4). *EF‐1* was used as an internal standard. Different letters indicate significant differences (Tukey's HSD, *p* < 0.05).

**FIGURE 5:**
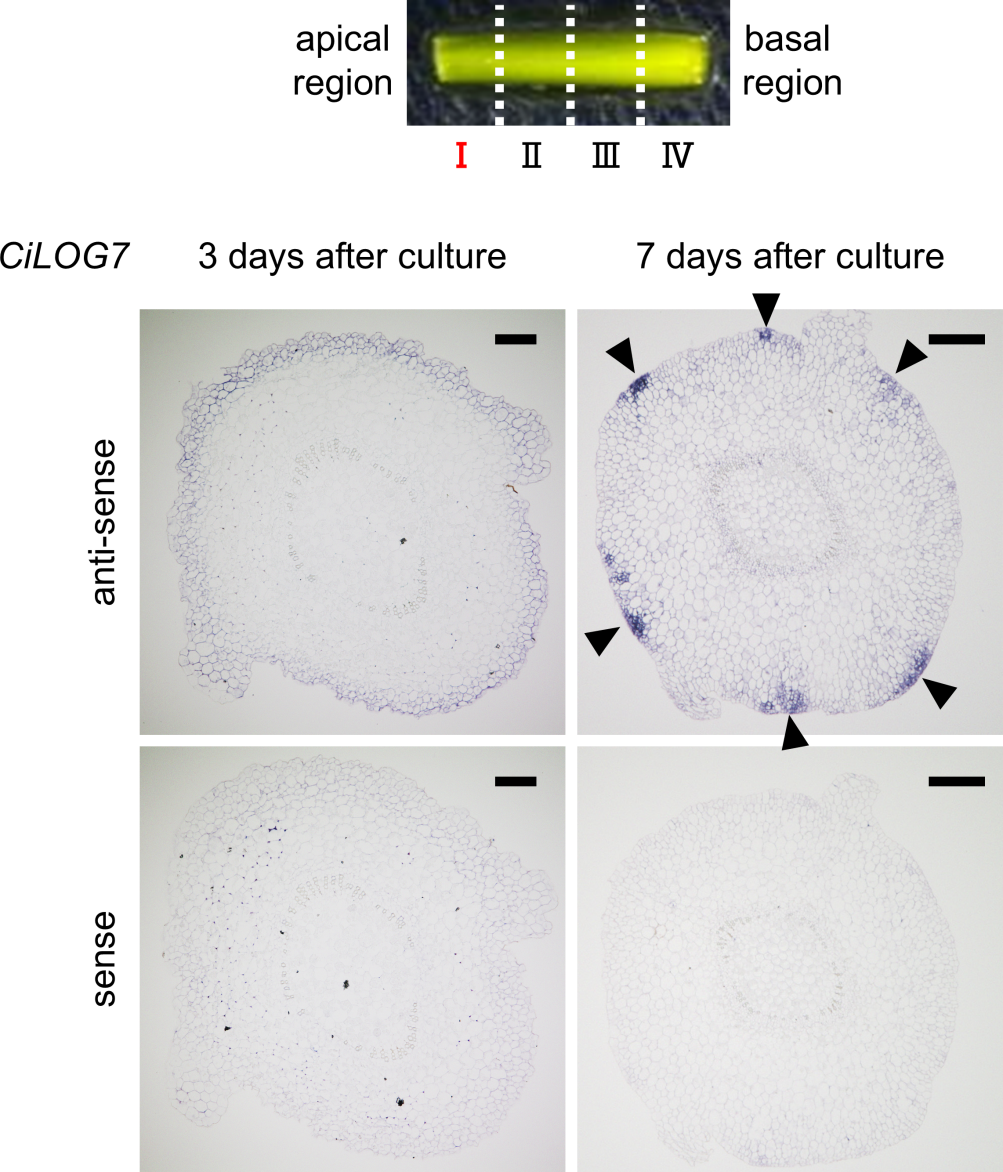
*In situ* hybridization analysis of *CiLOG7* on cross‐sections of internodal segments. The segments were cultured on a phytohormone‐free culture medium for 3 days or 7 days. The apical region (region I) was used. Arrowheads, *LOG7* expression in the epidermis after 7 days of culture. Bars, 200 μm.

After 2 or 3 days of culture, the pith, cortex, and epidermis regions were excised from cryo‐sections of the apical region by laser microdissection (Figure 4A), and the expression of *CiIPT3* and *CiLOG7* was analyzed by qRT‐PCR. *CiIPT3* expression was highest in the pith after 2 days of culture, whereas *CiLOG7* expression was highest in the epidermis after 3 days (Figure 4B). Among the four internodal segment regions, *CiLOG7* expression was highest in region I (Figure S4). Therefore, to investigate the localization of *CiLOG7* mRNA in the epidermis, we performed *in situ* hybridization in region I after 3 days and 7 days of culture (Figure 5). *CiLOG7* mRNA was present in the whole epidermis after 3 days, but its expression was detected as several spots on the epidermis after 7 days (Figure 5). We hypothesized that CK precursors were biosynthesized in the pith and transported to the epidermis, as *CiIPT3* and *CiLOG7* were expressed in different tissues. Therefore, internodal segments without pith were cultured on B5 medium containing CK precursors (1 μM iPR or 1 μM iPRP). Adventitious shoots formed on internodal segments without pith cultured on CK precursor‐containing culture medium from all segments tested (Figure S7).

### 3.5 Effects of auxin on CK biosynthesis gene expression and endogenous CK levels

**FIGURE 6:**
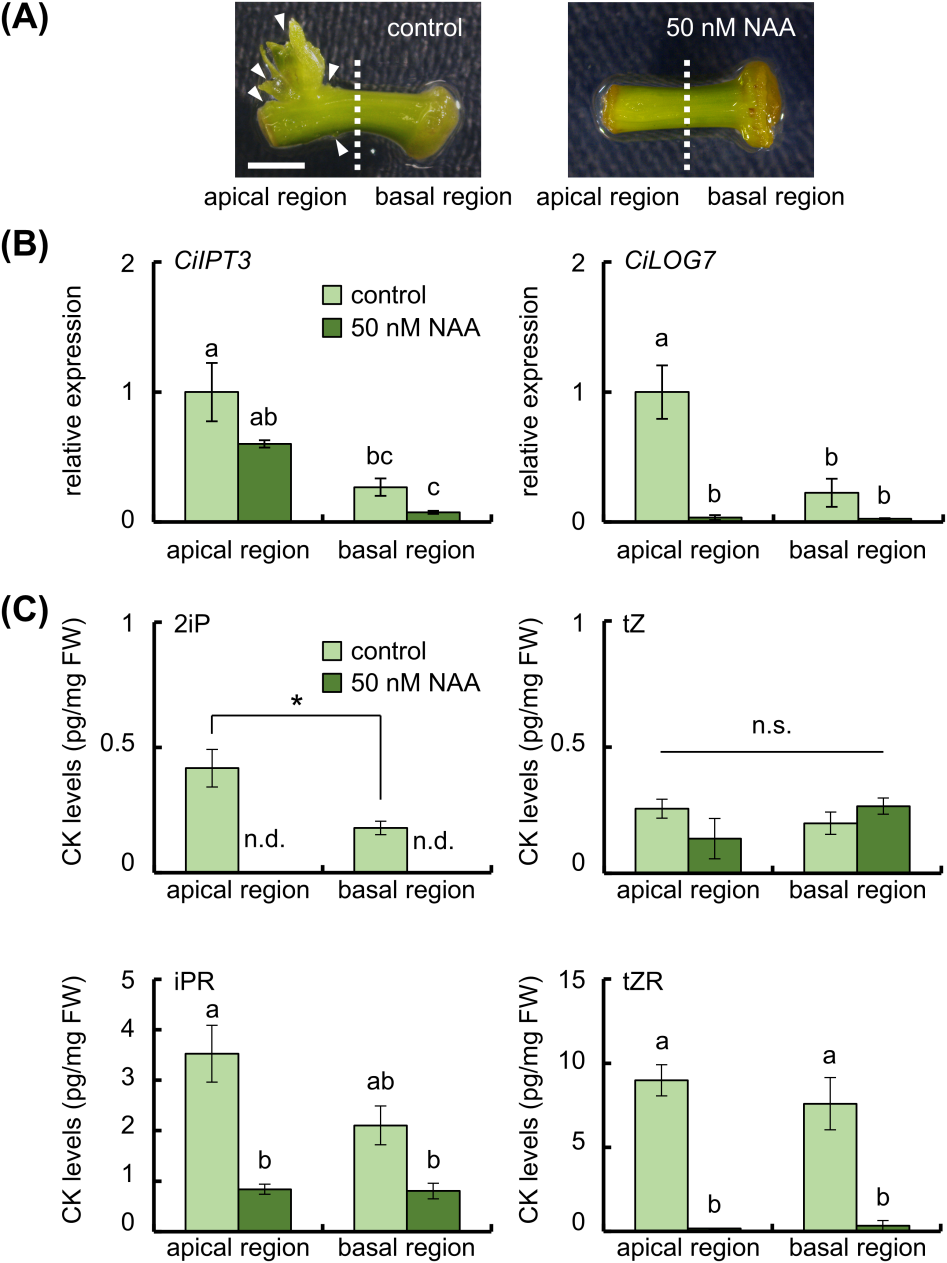
Effects of NAA treatment on CK biosynthesis in internodal segments. (A) Representative images of tissues after 35 days of culture under the indicated conditions. White arrowheads, adventitious shoots. Bar, 2 mm. (B) Relative expression of CK biosynthesis genes. *EF‐1* was used as an internal standard. Tissues were collected after 3 days of culture on a culture medium with or without 50 nM NAA. Then, the internodal segments were divided into the apical region and basal region sections. Five internodal segments were used in each experiment. (C) Endogenous CK levels in internodal segments. Data are mean ± SE (*n* = 3). Different letters indicate significant differences among segments (Tukey's HSD, *p* < 0.05, tZ, tZR, iPR). Asterisk indicates a significant difference compared with apical region (*t* test, *p* < 0.05). n.d.: not detected, n.s.: not significant.

Exogenously applied NAA strongly inhibited adventitious shoot formation in ipecac at low concertation (Figure 6A) (Koike et al. 2020). Therefore, we investigated the effects of NAA treatment on the expression of *CiIPT3* and *CiLOG7*. Treatment with 50 nM NAA significantly suppressed *CiLOG7* expression in the apical region of internodal segments but did not affect *CiIPT3* expression (Figure 6B). NAA treatment also decreased the endogenous levels of 2iP, iPR, and tZR, while tZ levels remained unchanged (Figure 6C). Notably, 2iP levels were under detection limit in both the apical and basal regions. In addition, an auxin antagonist, 4‐Cl‐PEO‐IAA, suppressed *CiLOG7* expression, decreased 2iP and iPR levels, and increased IAA levels in the apical region of internodal segments (Figure 8S).

## 4 DISCUSSION

In this study, we found that the pith of internodal segments is required for adventitious shoot formation in ipecac (Figure 1). The pith is also crucial for activation of endogenous CK biosynthesis in the internodal segments during culture (Figure 2). Upregulation of *CiIPT3* and *CiLOG7*, ipecac homologs of the *Arabidopsis IPT3* and *LOG7* genes, was confirmed in the apical region of internodal segments during culture (Okazaki et al. 2022). However, *CiIPT3* and *CiLOG7* exhibited distinct expression patterns, with *CiIPT3* expression increasing before *CiLOG7* expression (Figure 3). Furthermore, *CiIPT3* was expressed in the pith, whereas *CiLOG7* exhibited the highest expression in the epidermis (Figure 4), suggesting differential expression of these genes in different tissues of the internodal segments.

In *Arabidopsis*, nine *IPT* homologs encode enzymes for the first step of CK biosynthesis (Kakimoto 2001, 2003). ATP/ADP isopentenyl transferases AtIPT1 and AtIPT3–8 catalyze the transfer of an isopentenyl group from a dimethyl allyl pyrophosphate to ATP or ADP to produce 2iP ribotides, whereas tRNA isopentenyl transferases AtIPT2 and AtIPT9 catalyze isopentenylation of tRNA (Kakimoto 2003). *AtIPT3* is expressed in the phloem tissues of vascular bundles of both shoots and roots (Miyawaki et al. 2004). *CiIPT3* was expressed in the pith, including phloem tissues, of internodal segments in ipecac (Figure 4). *CiIPT3* expression and endogenous CK levels were reduced in the internodal segments without pith on phytohormone‐free media (Figure 2). Thus, the pith of internodal segments is needed for *CiIPT3* expression and start the biosynthesis of endogenous CKs in ipecac.

In *Arabidopsis*, nine homologs of *LOG* genes encode enzymes catalyzing the CK activation (Kuroha et al. 2009). *AtLOG7* is expressed in developing primordia of the shoot apical meristem (Yadav et al. 2009, Chickarmane et al. 2012) and is the most important gene in the maintenance of the size of this meristem (Tokunaga et al. 2012). *AtLOG7* mRNA is localized within the epidermis of the shoot apical meristem (the L1 layer) (Gruel et al. 2016). In ipecac internodal segments, *CiLOG7* was expressed mainly in the epidermis, not in the pith or cortex (Figure 4), and *in situ* hybridization revealed localized *CiLOG7* expression in distinct spots on the epidermis during culture (Figure 5), suggesting active CK production in the epidermis, leading to meristem development in adventitious shoots. The interval expression of *CiLOG7* might provide a “place” for the shoot growth because the shoot form becomes abnormal when multiple shoots are formed.

*Arabidopsis CYP735A*s are expressed mainly in roots and much more weakly in the hypocotyl and shoot apical region (Takei et al. 2004, Kiba et al. 2013). In intact *Arabidopsis* plants, tZ‐type CKs are produced mainly in roots, loaded into the xylem through ATP‐binding cassette transporter subfamily G14, and translocated from roots to shoots via the xylem (Ko et al. 2014, Zhang et al. 2014). When *Arabidopsis* hypocotyls are cultured on kinetin‐containing medium for *de novo* shoot regeneration, adventitious shoot formation is promoted by the kinetin treatment (Pernisova et al. 2018). In these explants, endogenous tZ‐type CK levels increase regardless of the kinetin concentration during culture, while endogenous 2iP‐type CK levels increase in response to kinetin treatment. Effects of endogenous 2iP were more efficient at the shoot regeneration than that of tZ. In ipecac, *CiCKH*, which is a CYP735A associated with tZ‐type‐CK biosynthesis, is not upregulated during the internode culture of ipecac (Figure 3). These data suggest that tZ‐type CK is not extensively biosynthesized in internodal segments. The levels of 2iP, iPR, and tZR increased mainly in the apical region, but tZ levels were low and did not differ between the apical and basal regions (Figure 3). In addition, tZ levels did not increase in the epidermis despite high *CiLOG7* expression (Figures 3 and 4). These results suggest that tZ ribotide does not move from pith and cortex to the epidermis. The position of adventitious shoot formation correlates with the localization of high 2iP levels, not tZ levels (Figure 3A,C). Taken together, these data suggest that 2iP‐type CKs play an important role in *de novo* shoot formation of ipecac.

*Torenia fournieri* also forms adventitious shoots on the epidermis of stem segments in the presence of exogenous CK (Tanimoto and Harada 1982). Following CK treatment, the epidermal cells transit from a mitotically inactive G1 or G0 phase to the S phase (Morinaka et al. 2021). In ipecac, the *CYCLIN D3* gene, which promotes G1‐to‐S phase transition, is upregulated in the apical region of internodal segments during culture (Okazaki et al. 2022). Endogenous CKs might initiate the cell cycle of epidermal cells to start the differentiation of shoot meristems in ipecac.

In addition, exogenously applied NAA strongly inhibited *CiLOG7* expression more than *CiIPT3* expression, resulting in reduced levels of 2iP, iPR, and tZR levels (Figure 6). Auxin suppresses adventitious shoot formation by suppressing ectopic *CiLOG7* expression. 4‐Cl‐PEO‐IAA specifically blocks the function of the auxin receptor TRANSPORT INHIBITOR RESPONSE 1 (TIR1) (Hayashi et al. 2012). Treatment with 4‐Cl‐PEO‐IAA also partially inhibits adventitious shoot formation in ipecac (Koike et al. 2020) and CK biosynthesis, leading to increased IAA levels in the apical region (Figure S8). The results suggest that IAA is produced through feedback regulation following 4‐Cl‐PEO‐IAA treatment; the increased IAA might bind to another auxin receptor, AUXIN BINDING PROTEIN 1, to suppress the *CiLOG7* expression.

Here, we propose a hypothetical model of adventitious shoot formation in ipecac (Figure S9). In the apical region of internodal segments, a CK precursor, isopentenyladenine riboside (iPRP), is produced from dimethyl allyl pyrophosphate and ATP/ADP by CiIPT3 in the pith of internodal segments. iPRP is transferred from the pith to the epidermis and is converted into the active CK 2iP by CiLOG7. 2iP accumulates locally in the epidermis and induces cell division to form adventitious shoots. Loss of the pith prevents adventitious shoot formation (Figures 1 and 2) because they no longer supply iPRP to the epidermis for the biosynthesis of active CKs. In contrast, auxin is transported from the apical region to the basal region through the polar auxin transport stream by PIN‐FORMED (PIN) transporters (Zazimalova et al. 2010). In ipecac, the IAA levels decreased after internodal segments were cut off, and IAA in the apical region remained lower than that in the basal region because IAA was transported from the former to the latter, resulting in callus formation in the basal region (Koike et al. 2020) (Figure S5). In the basal region, increased IAA suppresses adventitious shoot formation by suppression of *CiLOG7* expression. It is difficult to compare our results with those of other studies directly because of the difference in experimental conditions, but the CK‐to‐auxin ratio is much higher in ipecac than in *Arabidopsis* and tomato (Ikeuchi et al. 2017, Iwase et al. 2018, Larriba et al. 2021). An increase in this ratio creates conditions suitable for adventitious shoot formation in the apical region of internodal segments. After the increase in endogenous CK levels, the acquisition of cellular pluripotency and the initiation of cell division would be induced for the adventitious shoot formation. Future investigations will focus on understanding the regulation of *CiIPT3* and *CiLOG7* transcription during adventitious shoot formation in ipecac.

## AUTHOR CONTRIBUTIONS

K.O. and M.U. conceived and designed the research. Ko.S. provided sterile ipecac plants. K.O. conducted the ipecac tissue culture experiments. K.O., Ky.S., and M.A. conducted lazer microdissection and gene expression analysis. K.O., W.K., and K.K.T. conducted *in situ* hybridization. K.O. and M.U. analyzed the data. K.O., M.A., K.K.T, Ko.S., and M.U. wrote the manuscript. All authors read and approved the manuscript.

## FUNDING INFORMATION

This work was in part supported by the Inoue Enryo Memorial Foundation for Promoting Science from Toyo University to KO, the Sasagawa Scientific Research Grant from The Japan Science Society to KO (2022–4085), JST SPRING to KO (JPMJSP2159), Grant‐in‐Aid for JSPS Fellows from the Japan Society for the Promotion of Science to KO (23KJ1980), the PLANTX bridge fellowship from PLANTX Corporation to KO, the Program for Promotion of Practical Use of Intellectual Property from Toyo University to MU, and the 30th, 31st, and 32nd Botanical Research Grants from Ichimura Foundation for New Technology to MU.

## Supporting information


**Figure S1.** Sudan Red 7B staining of ipecac internodal segments with *de novo* shoots and *Arabidopsis* zygotic embryos. Bars, 1 mm.
**Figure S2** Cryo‐sections of internodal segments with or without the pith including vascular bundles. The sections were stained with toluidine blue *O*. Arrowheads, pith. Bars, 200 μm.
**Figure S3** Effects of internodal segment length on adventitious shoot formation. (A) Representative images of internodal segments with adventitious shoots after 5 weeks of culture on a phytohormone‐free culture medium. Bar, 1 mm. The length of each segment is indicated. (B) Total number of adventitious shoots formed on an internodal segment. (C) Number of adventitious shoots in the apical and basal regions of the internodal segments after 5 weeks of culture. Ten segments were used in each experiment. Data are means ± SE (*n* = 4). Different letters indicate significant differences (Tukey's HSD, *p* < 0.05).
**Figure S4** Relative expression of CK biosynthesis genes in internodal segments. Each segment (5 mm) was cut into four sections (apical to basal, I to IV) before culture (0 day) or after 7 days of culture on a phytohormone‐free culture medium. Eight segments were used in each experiment. Data are means ± SE (*n* = 4). *EF‐1* was used as an internal standard. Different letters indicate significant differences among segments (Tukey's HSD, *p* < 0.05).
**Figure S5** Endogenous IAA levels in internodal segments. (A) Internodal segments (4 with and 14 without the pith) were used in each experiment. They were collected before culture (0 day) or after 7 days of culture. Data are means ± SE (*n* = 4). (B) Time course analysis of endogenous IAA in internodal segments during the initial 7 days of culture. The segments were collected before culture (0 day) or after 1 h, 3 h, 6 h, 12 h, 1 day, 2 days, 3 days, 5 days, and 7 days of culture. Inset shows an enlarged 0–6‐h plot. Four segments were used in each experiment. Data are means ± SE (*n* = 3). Asterisk indicates significant difference compared with apical region (*t‐*test, *p* < 0.05).
**Figure S6** Time course analysis of a CK metabolism gene (*CiCKX7*) in internodal segments during the initial 7 days of culture. *EF‐1* was used as an internal standard. The data were normalized to the *CiCKX7* expression in the apical region before culture. Data are means ± SE (*n* = 4). Internodal segments were collected before culture (0 day), after 1 h, 3 h, 6 h, 12 h, 1 day, 2 days, 3 days, 5 days, and 7 days of culture on phytohormone‐free culture medium. Then, the internodal segments were divided into the apical region and basal region sections. Five segments were used in each experiment. Asterisk indicates significant difference compared with apical region (*t*‐test, *p* < 0.05).
**Figure S7** Effects of CK precursors on adventitious shoot formation in ipecac. Representative images of tissues after 4 weeks of culture under the indicated conditions. White arrowheads, adventitious shoots. Number of internodal segments with adventitious shoots formed are shown in each image. The experiment was conducted twice, with six segments per experiment. 2iP, *N* ^6^‐isopentenyladenine. iPR, *N* ^6^‐isopentenyl adenine riboside. iPRP, *N* ^6^‐isopentenyladenine‐9‐riboside‐5′ phosphate. Bar, 2 mm.
**Figure S8** Effects of 4‐Cl‐PEO‐IAA treatment on CK and IAA biosynthesis in internodal segments. (A) Relative expression of CK biosynthesis genes. *EF‐1* was used as an internal standard. Tissues were collected after 3 days of culture on a culture medium with or without 10 μM 4‐Cl‐PEO‐IAA. Then, the internodal segments were divided into the apical region and basal region sections. Five internodal segments were used in each experiment. (B) Endogenous CK and auxin levels in internodal segments. Data are means ± SE (*n* = 3). Asterisk indicates significant difference compared with apical region (*t* test, *p* < 0.05). n.s.: not significant.
**Figure S9** A hypothetical model on distribution of endogenous phytohormones during adventitious shoot formation in ipecac. Dotted lines indicate the estimated flow in internodal segments.

## Data Availability

All data supporting the findings of this work (including supplementary materials) are available from the corresponding author upon request.
