# Supplementary material for: Ectopic expression of LONELY GUY7 in epidermis of internodal segments for de novo shoot regeneration without phytohormone treatment in ipecac
Source: Physiol Plant. 2024 Dec 26;177(1):e70023. doi: 10.1111/ppl.70023 (PMC11670440; doi:10.1111/ppl.70023)
Supplement: Supplementary file 1 — Figure S1. Sudan Red 7B staining of ipecac internodal segments with de novo shoots and Arabidopsis zygotic embryos. Bars, 1 mm. Figure S2 Cryo‐sections of internodal segments with or without the pith including vascular bundles. The sections were stained with toluidine blue O. Arrowheads, pith. Bars, 200 μm. Figure S3 Effects of internodal segment length on adventitious shoot formation. (A) Representative images of internodal segments with adventitious shoots after 5 weeks of culture on a phytohormone‐free culture medium. Bar, 1 mm. The length of each segment is indicated. (B) Total number of adventitious shoots formed on an internodal segment. (C) Number of adventitious shoots in the apical and basal regions of the internodal segments after 5 weeks of culture. Ten segments were used in each experiment. Data are means ± SE (n = 4). Different letters indicate significant differences (Tukey's HSD, p < 0.05). Figure S4 Relative expression of CK biosynthesis genes in internodal segments. Each segment (5 mm) was cut into four sections (apical to basal, I to IV) before culture (0 day) or after 7 days of culture on a phytohormone‐free culture medium. Eight segments were used in each experiment. Data are means ± SE (n = 4). EF‐1 was used as an internal standard. Different letters indicate significant differences among segments (Tukey's HSD, p < 0.05). Figure S5 Endogenous IAA levels in internodal segments. (A) Internodal segments (4 with and 14 without the pith) were used in each experiment. They were collected before culture (0 day) or after 7 days of culture. Data are means ± SE (n = 4). (B) Time course analysis of endogenous IAA in internodal segments during the initial 7 days of culture. The segments were collected before culture (0 day) or after 1 h, 3 h, 6 h, 12 h, 1 day, 2 days, 3 days, 5 days, and 7 days of culture. Inset shows an enlarged 0–6‐h plot. Four segments were used in each experiment. Data are means ± SE (n = 3). Asterisk indicates significant difference co [file PPL-177-e70023-s001.pdf]

Figure S1

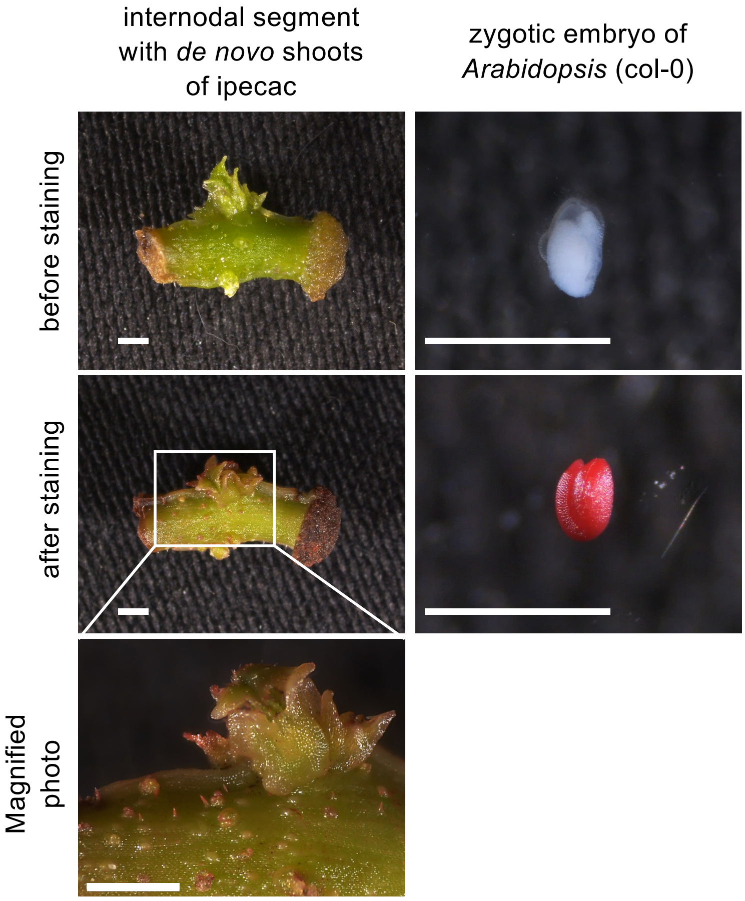

Figure S1 Sudan Red 7B staining of ipecac internodal segments with *de novo* shoots and *Arabidopsis* zygotic embryos. Bars, 1 mm.

Figure S2

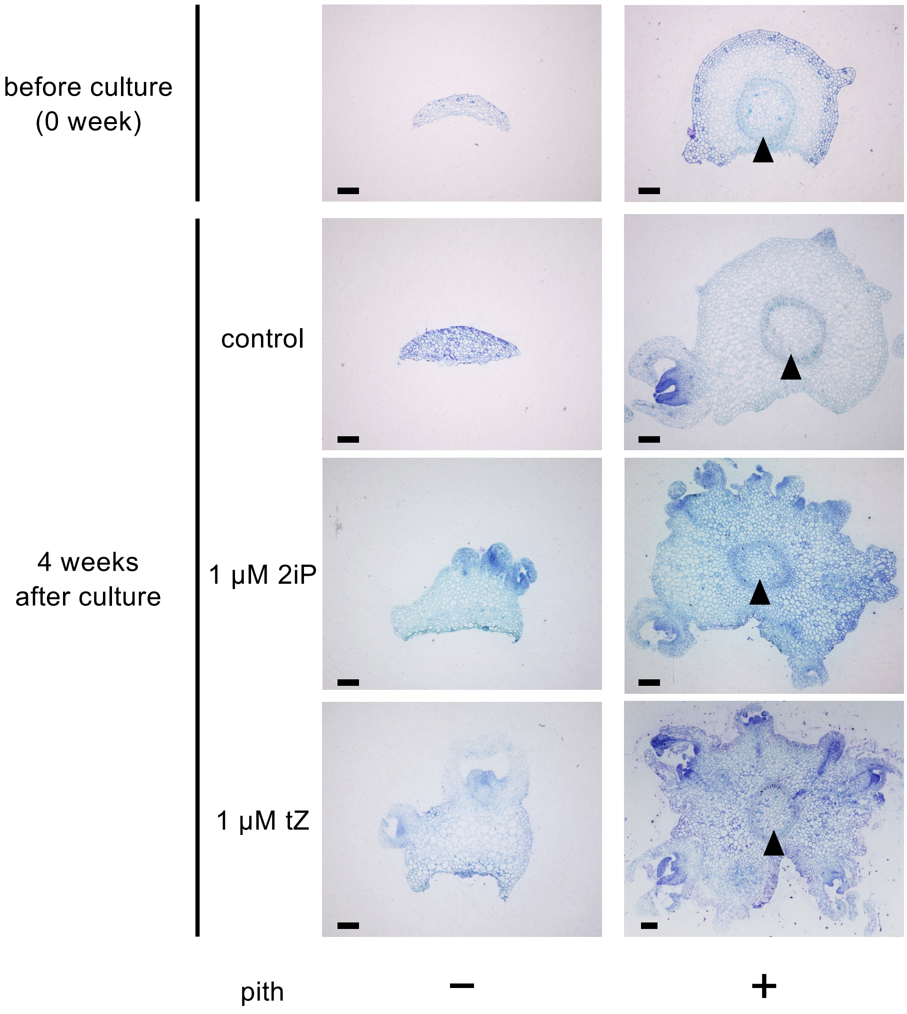

Figure S2 Cryo-sections of internodal segments with or without the pith including vascular bundles. The sections were stained with toluidine blue *O*. Arrowheads, pith. Bars, 200 μm.

Figure S3

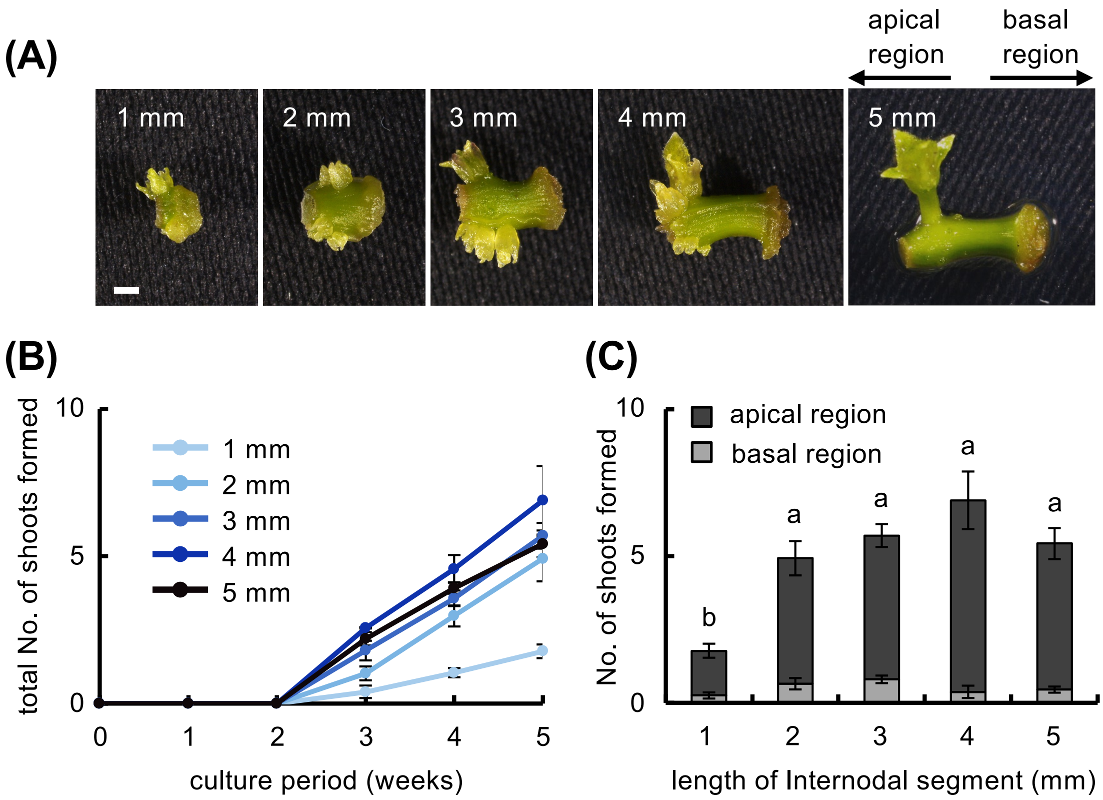

Figure S3 Effects of internodal segment length on adventitious shoot formation. (A) Representative images of internodal segments with adventitious shoots after 5 weeks of culture on a phytohormone-free culture medium. Bar, 1 mm. The length of each segment is indicated. (B) Total number of adventitious shoots formed on an internodal segment. (C) Number of adventitious shoots in the apical and basal regions of the internodal segments after 5 weeks of culture. Ten segments were used in each experiment. Data are means ± SE (n = 4). Different letters indicate significant differences (Tukey's HSD, $P < 0.05$).

Figure S4

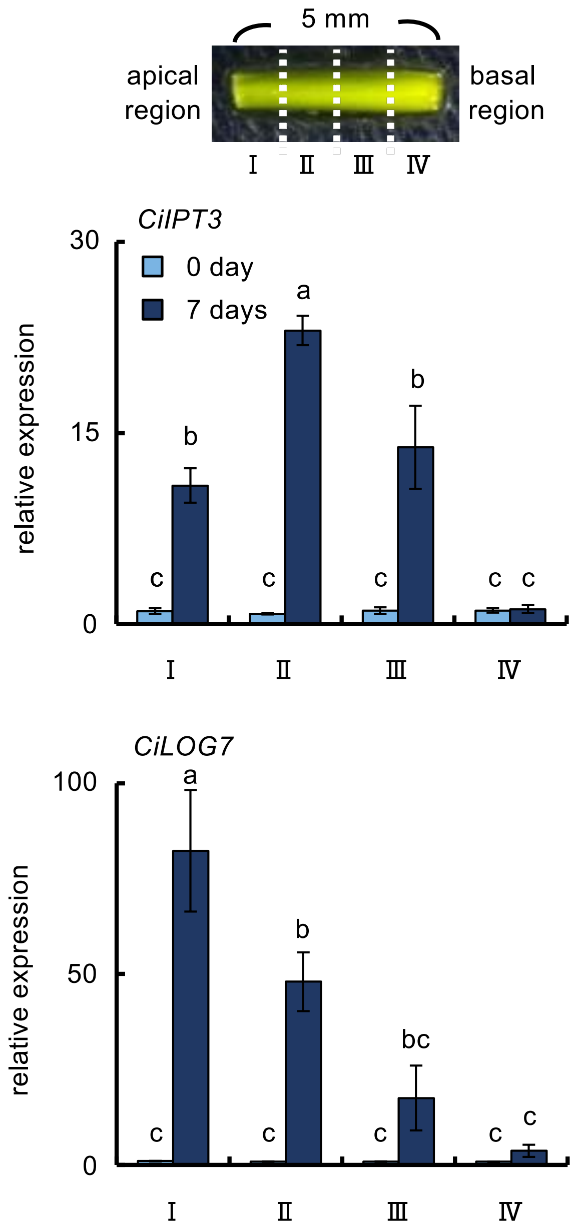

Figure S4 Relative expression of CK biosynthesis genes in internodal segments. Each segment (5 mm) was cut into four sections (apical to basal, Ⅰ to Ⅳ) before culture (0 day) or after 7 days of culture on a phytohormone-free culture medium. Eight segments were used in each experiment. Data are means ± SE (n = 4). *EF-1* was used as an internal standard. Different letters indicate significant differences among segments (Tukey's HSD, $P < 0.05$).

Figure S5

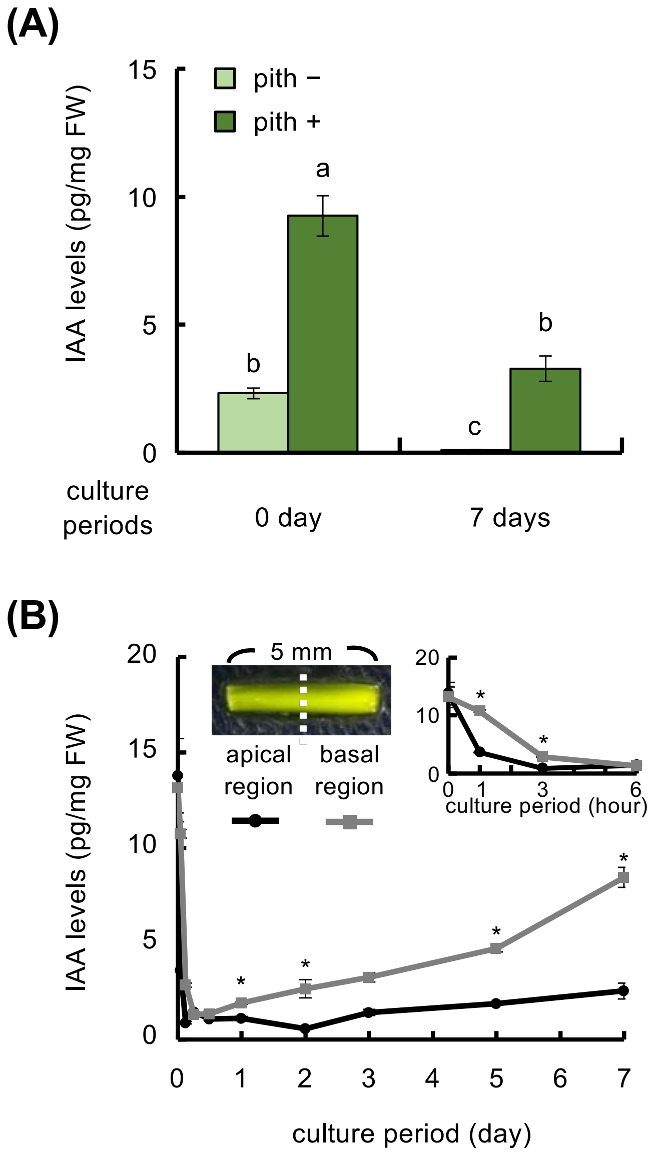

Figure S5 Endogenous IAA levels in internodal segments. (A) Internodal segments (4 with and 14 without the pith) were used in each experiment. They were collected before culture (0 day) or after 7 days of culture. Data are means ± SE (*n* = 4). Different letters indicate significant differences (Tukey's HSD, $P < 0.05$). (B) Time course analysis of endogenous IAA in internodal segments during the initial 7 days of culture. The segments were collected before culture (0 day) or after 1 h, 3 h, 6 h, 12 h, 1 day, 2 days, 3 days, 5 days, and 7 days of culture. Inset shows an enlarged 0–6-h plot. Four segments were used in each experiment. Data are means ± SE (*n* = 3). Asterisk indicates significant difference compared with apical region (*t*-test, $P <$ 0.05).

Figure S6

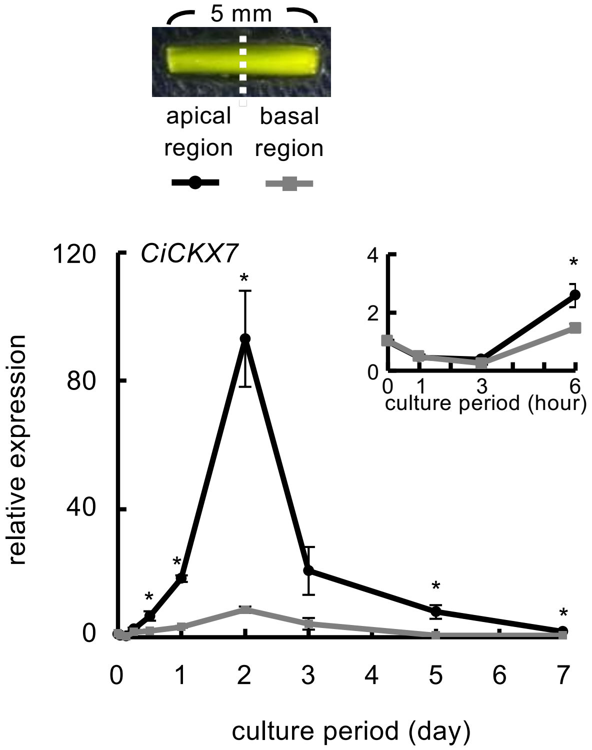

Figure S6 Time course analysis of a CK metabolism gene (*CiCKX7*) in internodal segments during the initial 7 days of culture. *EF-1* was used as an internal standard. The data were normalized to the *CiCKX7* expression in the apical region before culture. Data are means ± SE (n = 4). Internodal segments were collected before culture (0 day), after 1 h, 3 h, 6 h, 12 h, 1 day, 2 days, 3 days, 5 days, and 7 days of culture on phytohormone-free culture medium. Then, the internodal segments were divided into the apical region and basal region sections. Five segments were used in each experiment. Asterisk indicates significant difference compared with apical region (t-test, $P < 0.05$).

Figure S7

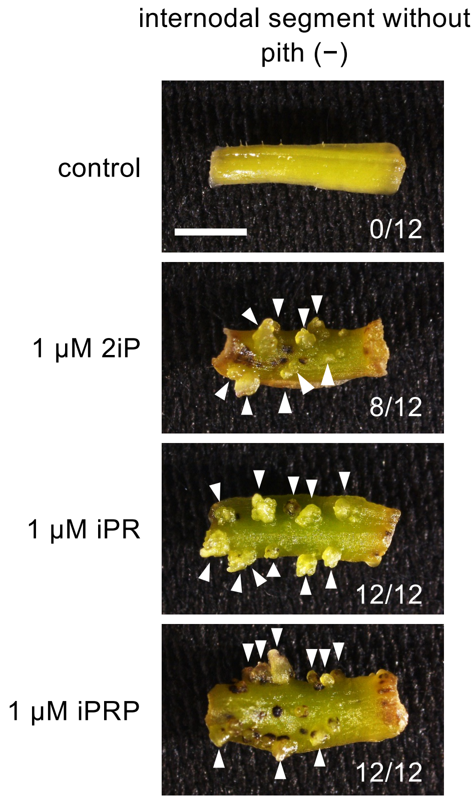

Figure S7 Effects of CK precursors on adventitious shoot formation in ipecac. Representative images of tissues after 4 weeks of culture under the indicated conditions. White arrowheads, adventitious shoots. Number of internodal segments with adventitious shoots formed are shown in each image. The experiment was conducted twice, with six segments per experiment. 2iP, *N*6-isopentenyladenine. iPR, *N*6-isopentenyl adenine riboside. iPRP, *N*6-isopentenyladenine-9-riboside-5′ phosphate. Bar, 2 mm.

Figure S8

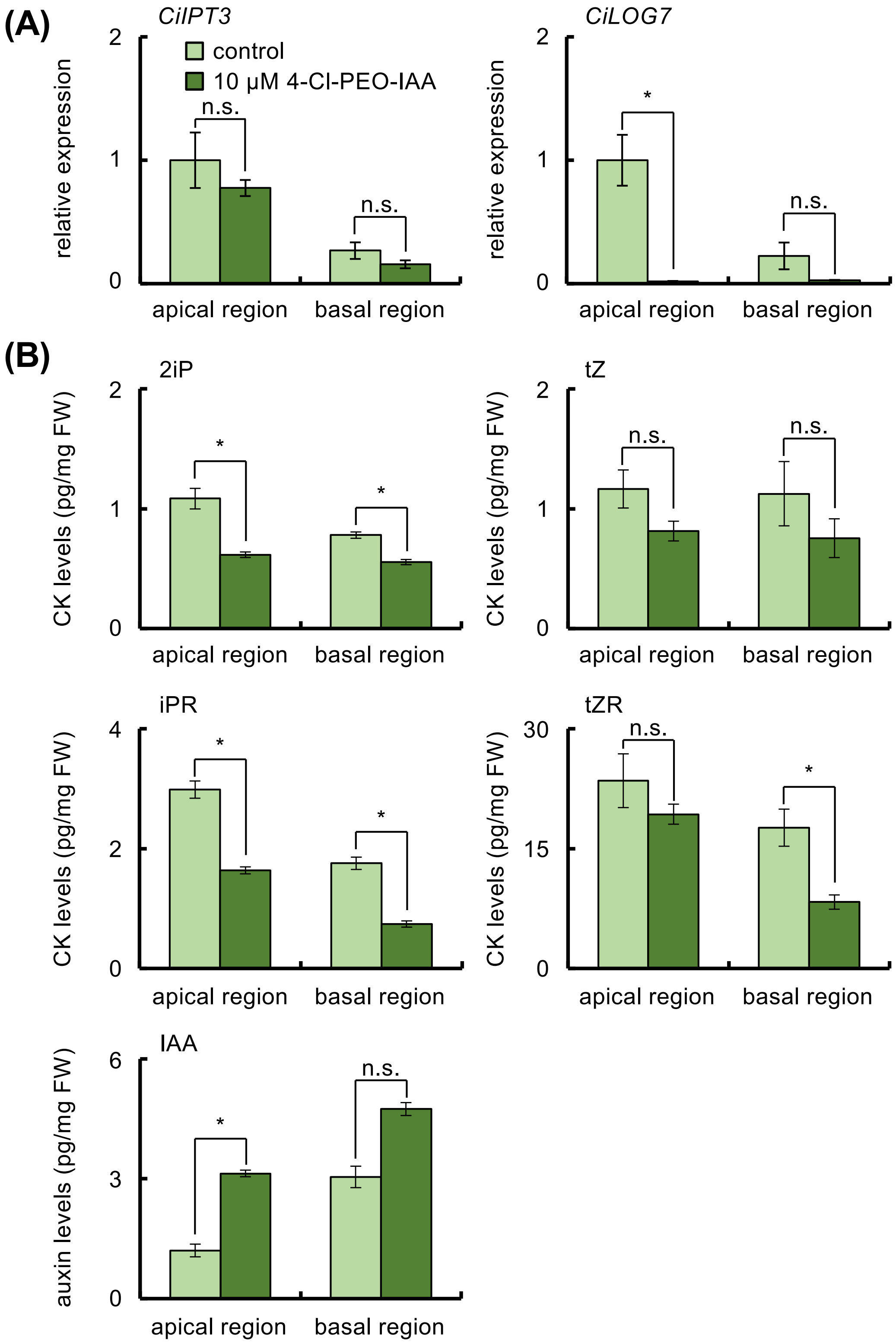

Figure S8 Effects of 4-Cl-PEO-IAA treatment on CK and IAA biosynthesis in internodal segments. (A) Relative expression of CK biosynthesis genes. *EF-1* was used as an internal standard. Tissues were collected after 3 days of culture on a culture medium with or without 10 μM 4-Cl-PEO-IAA. Then, the internodal segments were divided into the apical region and basal region sections. Five internodal segments were used in each experiment. (B) Endogenous CK and auxin levels in internodal segments. Data are means ± SE (n = 3). Asterisk indicates significant difference compared with apical region (t-test, P < 0.05). n.s.: not significant.

Figure S9

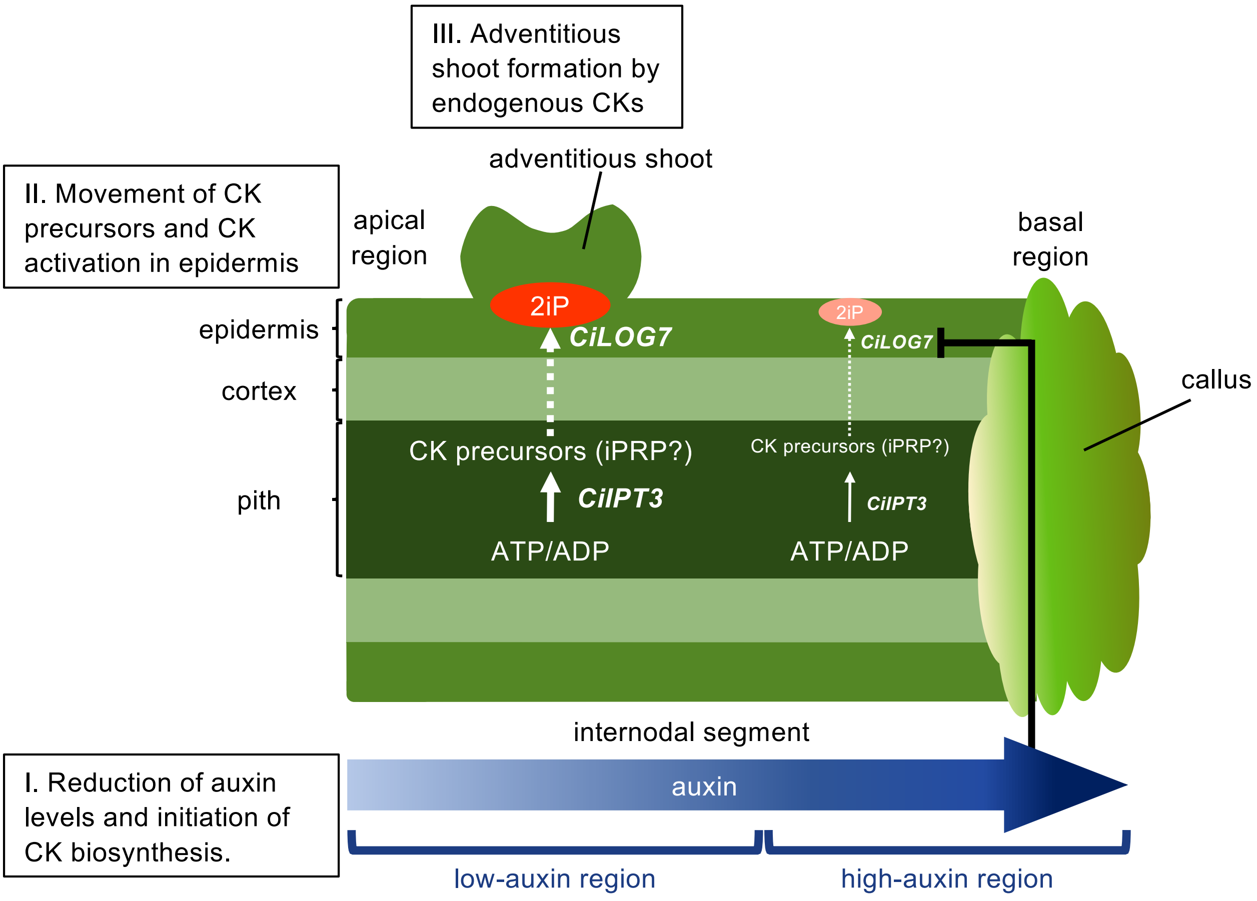

Figure S9 A hypothetical model on distribution of endogenous phytohormones during adventitious shoot formation in ipecac. Dotted lines indicate the estimated flow in internodal segments.

Table S1 Primers used in this study.

primer name		5'-Sequence-3'
CiEF1	F*	ACGGAGCAACCAAGAGAAGA
for qRT-PCR	R**	GTGGCTTCACCGATCAAGTT
CiIPT3	F	TCGGGGTAAGCTTCCAATCATC
for qRT-PCR	R	GAATTGAAACGTCCACCCACAG
CiLOG7	F	TGGGTCTGATGGGTCTTGTTTC
for qRT-PCR	R	ACCGGTTATCTCTCTTGGCATG
CiCKH	F	TCAAGAACGACAGACAGCCC
for qRT-PCR	R	CATCGTCATCCATCGCTCCA
CiCKX7	F	GCTGCCCAAAAATGGTGAGG
for qRT-PCR	R	CGGGTCATCACTGTTCACGA
CiLOG7	F	CGGGCTGCAGGAATTATGGAGGAAGAGAGAAAATCAAGGT
for probes	R	GCTTGATATCGAATTTTAAATAGGAACTTCAGTCTCTGGTG

*F: forward primer, **R: reverse primer
